# Clinical Evidence for the Microbiome in Inflammatory Diseases

**DOI:** 10.3389/fimmu.2017.00400

**Published:** 2017-04-12

**Authors:** Ann E. Slingerland, Zaker Schwabkey, Diana H. Wiesnoski, Robert R. Jenq

**Affiliations:** ^1^Immunology Program, Memorial Sloan Kettering Cancer Center, New York, NY, USA; ^2^Department of Genomic Medicine, Division of Cancer Medicine, University of Texas MD Anderson Cancer Center, Houston, TX, USA; ^3^Department of Stem Cell Transplantation Cellular Therapy, Division of Cancer Medicine, University of Texas MD Anderson Cancer Center, Houston, TX, USA

**Keywords:** microbiome, inflammation, bacteria, autoimmune diseases, microbiota

## Abstract

Clinical evidence is accumulating for a role of the microbiome in contributing to or modulating severity of inflammatory diseases. These studies can be organized by various organ systems involved, as well as type of study approach utilized, whether investigators compared the microbiome of cases versus controls, followed patients longitudinally, or intervened with antibiotics, prebiotics, or bacterial introduction. In this review, we summarize the clinical evidence supporting the microbiome as an important mechanism in the onset and maintenance of inflammation.

## Introduction

The human body is host to hundreds of thousands of bacteria and other microorganisms, primarily colonizing our epithelial surfaces and attaining their highest densities within the lower gastrointestinal tract. These commensals play an important homeostatic role in a variety of our body systems, including the immune system, and can have both immune-stimulatory and immune-regulatory effects. Intriguingly, microbiota differences have been associated with a variety of inflammatory diseases, indicating that targeting or modulating the microbiota may be a novel therapeutic strategy that could nicely complement established treatments for inflammatory conditions.

At the extremes of age, humans show changes in microbiome composition. The degree of diversity observed in the fecal microbiota is highest at 2 years of age, and the gut microbiota undergoes dramatic changes during the first 3 years of life, after which it stabilizes and generally changes only slightly throughout adulthood (1). This pattern of early fluctuations in life followed by stabilization has been attributed to dietary changes as infants transition from breastfeeding or formula to more solid food (2).

After birth, the mode of delivery has been seen to impact on the microbial composition of the newborn, with infants born vaginally exhibiting bacterial communities of their own mother’s vaginal microbiota, while infants born by cesarean section showed bacterial communities of skin origin (1). Moreover, the intestinal microbiota of the mother can shape the bacterial colonization of their infant’s gut (3). Even the placenta seems to have a unique microbiome that correlates with those found in the oral cavity (4). This may raise a question whether these findings correlate with higher chances of preterm deliveries in mothers with periodontal diseases (5) and modulating the oral microbiota could potentially prevent such complications during pregnancy.

The Human Microbiome Project was a NIH initiative launched in 2008 on the heels of the Human Genome Project that reflected an increasing interest in the study of microbes as complex and dynamic communities rather than isolated species. The interdisciplinary collaboration set out to characterize the human microbiome and explore the relationship between population fluctuations and disease; to establish new computational tools and strategies to complement a resource repository for ongoing studies; and to study the ethical, legal, and social implications of the field’s expansion. The 5-year initiative accelerated the progress of what had been a nascent discipline and facilitated our in-depth and multi-omic understanding of human microbiome science (6).

This review summarizes clinical studies that have helped to elucidate the involvement of the microbiota in inflammatory diseases. In addition to describing findings, we have also made an effort to provide context regarding numbers of patients and types of studies performed in Table 1 to allow the reader to better gage the quality of evidence being presented.

**Table 1:** **Clinical studies of microbiome associations and interventions in inflammatory conditions**.

Condition	Longitudinal cohort studies	Case–control studies	Cross-sectional studies	Interventional
Inflammatory bowel diseases		(14) (*n* = 227)		(61) (*n* = 31)
Ulcerative colitis				(60) (*n* = 59), (64) (*n* = 19), (66) (*n* = 29), (67) (*n* = 105), (17) (*n* = 120), (18) (*n* = 327), (19) (*n* = 120), (20) (*n* = 100), (21) (*n* = 90), (22) (*n* = 40), (23) (*n* = 26), (24) (*n* = 15), (26) (*n* = 29), (27) (*n* = 90), (29) (*n* = 144), (28) (*n* = 147), (30) (*n* = 40), (31) (*n* = 20), (32) (*n* = 30), (33) (*n* = 30), (34) (*n* = 32), (68) (*n* = 18), (69) (*n* = 41), (76) (*n* = 9), (77) (*n* = 11), (79) (*n* = 11), (80) (*n* = 8), (81) (*n* = 6), (82) (*n* = 5), (83) (*n* = 6), (88) (*n* = 3), (84) (*n* = 62), (89) (*n* = 75), (90) (*n* = 48), (78) (*n* = 1), (85) (*n* = 1)
Pouchitis				(65) (*n* = 20), (43) (*n* = 12), (45) (*n* = 20), (36) (*n* = 40), (38) (*n* = 40), (37) (*n* = 36), (39) (*n* = 31), (41) (*n* = 43), (42) (*n* = 16), (40) (*n* = 31)
Crohn’s disease		(15) (*n* = 38), (16) (*n* = 21)		(59) (*n* = 22), (62) (*n* = 10), (63) (*n* = 103), (46) (*n* = 20), (47) (*n* = 32), (48) (*n* = 34), (49) (*n* = 165), (50) (*n* = 120), (54) (*n* = 119), (55) (*n* = 30), (52) (*n* = 11), (53) (*n* = 75), (51) (*n* = 37), (56) (*n* = 28), (57) (*n* = 98), (58) (*n* = 70), (70) (*n* = 30), (71) (*n* = 10), (72) (*n* = 4), (73) (*n* = 30), (74) (*n* = 4), (75) (*n* = 5), (86) (*n* = 8), (87) (*n* = 19)
Multiple sclerosis		(132) (*n* = 78), (133) (*n* = 67), (134) (*n* = 40), (135) (*n* = 15), (136) (*n* = 103), (137) (*n* = 24), (139) (*n* = 61)	(138) (*n* = 17)	(141) (*n* = 12), (142) (*n* = 5), (140) (*n* = 3)
Guillain–Barré syndrome		(145) (*n* = 171), (146) (*n* = 103), (147) (*n* = 212), (148) (*n* = 549)	(144) (*n* = 56)	
Fibromyalgia		(151) (*n* = 95), (152) (*n* = 114), (154) (*n* = 100), (155) (*n* = 233), (156) (*n* = 1,125), (157) (*n* = 251), (158) (*n* = 168)	(153) (*n* = 233)	
Systemic lupus erythematosus		(161) (*n* = 40), (162) (*n* = 35)		
Atherosclerosis	(98) (*n* = 2,595), (99) (*n* = 4,007)	(92) (*n* = 17), (91) (*n* = 2), (93) (*n* = 49), (96) (*n* = 7)		
Vasculitis		(94) (*n* = 38), (95) (*n* = 31), (100) (*n* = 17)		
Eczema		(103) (*n* = 23)		
Scleroderma		(112) (*n* = 4), (110) (*n* = 80)		
Psoriasis		(106) (*n* = 6), (107) (*n* = 22), (108) (*n* = 9,295)		
Rheumatoid arthritis		(114) (*n* = 30), (117) (*n* = 83), (116) (*n* = 156), (118) (*n* = 114), (113) (*n* = 212)		(115) (*n* = 46)
Asthma	(121) (*n* = 321), (124) (*n* = 411), (125) (*n* = 47), (126) (*n* = 319)	(120) (*n* = 43), (122) (*n* = 20), (123) (*n* = 51)		

## Strategies of Gut Microbiota Modulation

**Figure 1:**
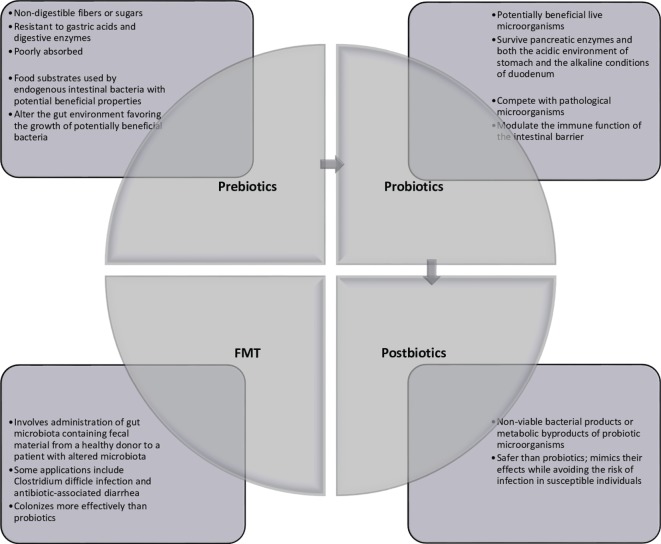
**Current strategies of gut microbiota modulation**.

Multiple avenues for therapeutic modulation of the microbiota have been gleaned from the continued research into microbiota–host interactions. Most widely administered are probiotics, which can be administered by way of lyophilized or live single or combination of bacterial preparations or fermented foodstuffs (primarily milk products) containing known active cultures. The benefits of both single-strain preparations and synergistic bacterial mixtures are loosely attributed to any of three mechanisms: (1) interference with the growth or survival of pathogenic microorganisms in the gut lumen, (2) improvement of mucosal barrier function or mucosal immune system, and (3) influence beyond the gut through the systemic immune system and other organs (7). Administration of prebiotics, which are non-digestible food ingredients, is designed to enrich native beneficial populations. While a number of dietary carbohydrates show promise for such use, not all have been formally evaluated to meet criteria for classification as a prebiotic compound. Such ingredients must (1) be neither hydrolyzed nor absorbed in the upper digestive tract, thus ensuring fermentation in the colon, (2) be a selective substrate for specific potentially beneficial commensal bacteria in the colon, stimulating growth and expansion or metabolic activation, and (3) thus be capable of effecting beneficial shifts in colonic bacterial communities (8). Live microbes are also administered with prebiotic substrates in preparations known as synbiotics. In addition to conferring the benefits of both probiotics and prebiotics, synbiotics are thought to improve the survival of the probiotic organism in the host by making its specific fermentable substrate readily available (9). In contrast, modulation of the intestinal microbiota has also been achieved through selective decontamination with narrow-spectrum antimicrobials. Removal or attenuation of undesirable bacterial species allows for reorganization of other populations within existing ecological niches (10). Newer treatment modalities include fecal microbiota transplant (FMT), introducing the complete microbial ecology of a healthy donor to restore phylogenetic diversity and synergistic function (11), and administration of bacterial metabolic byproducts such as short-chain fatty acids or conjugated bile acids (12). These strategies are summarized in Figure 1.

## Gastrointestinal–Inflammatory Bowel Diseases (IBD)

The gastrointestinal tract includes several sites that harbor the highest densities of bacterial commensals, and thus much of the exploration of the potential of therapeutic modulation of the intestinal microbiota has been focused on patients with disorders of the digestive system. In inflammatory bowel disease (IBD), comprised largely by Crohn’s disease (CD) and ulcerative colitis (UC), genetic susceptibilities are thought to predispose to development of heightened immunity against enteral “dysbiosis,” a term used to describe a deviation or perturbation from a healthy microbiome (13). In patients with UC, inflammation is localized primarily to the colonic mucosa, while in CD, discrete portions of the entire gastrointestinal tract can be involved, particularly the terminal ileum. Currently, no specific microorganism has been implicated in the pathogenesis or pathophysiology of IBD, but differences in the abundance and biodiversity of the enteric flora of individuals affected by IBD have been observed. Twin studies have elucidated the role of the microbiota excluding genetic involvement and explored potential environmental triggers of disease onset. An examination of twin pairs with discordance in their disease status found that risk factors for IBD included early gastrointestinal infection, implicating a potential role for different subsets of intestinal bacteria in maintaining or disturbing homeostasis of the gastrointestinal tract (14). Furthermore, compared to healthy and concordant twin pairs, discordant pairs were more likely to have disparities in microbiome parameters (15). An analysis of the mucosa-associated microbiota of CD patients identified that a reduction of Firmicutes member *Faecalibacterium prausnitzii* was associated with a higher risk of endoscopic and surgical disease recurrence (16). *F. prausnitzii* supernatant also reduced the transcription and secretion of pro-inflammatory molecules and mitigated symptom severity in murine colitis models both *in vitro* and *in vivo*.

### Probiotics and UC

There is a sizeable body of research examining the clinical effects of introducing single bacterial preparations and probiotic mixtures to patients with IBD, overall with variable results on disease outcomes. The oral probiotic strain *Escherichia coli* Nissle 1917 (EcN) was found to be equivalent over both short-term and long-term periods to standard 5-aminosalicylic acid (5-ASA) treatment for UC in three controlled studies (17–19), but had no benefit when used as an adjunct to ciprofloxacin treatment (20). In a double-blind study of 90 patients with UC, EcN enemas led to no changes in remission rates of UC between the groups, although there was a dose-dependent trend where patients who received a higher volume of daily EcN enemas responded best (21). A number of other single probiotic species have been studied for their ability to induce UC remission, with varying results. Rectal administration of *Lactobacillus reuteri* had a significant effect on mucosal cytokines, including increased IL-10 secretion leading to remission in 31% of pediatric subjects (22). In contrast neither rectal nor oral administration of *Lactobacillus casei* resulted in improvement in clinical activity scores following a 2-month trial, although there was a decrease in inflammatory cytokine activity and increased secretion of IL-10 seen with oral *L. casei* (23).

Administration of probiotic product VSL#3, a cocktail of eight different bacteria (four species of lactobacilli, three species of bifidobacteria, and *Streptococcus thermophilus*) has been quite extensively evaluated in patients with UC and found to have some promise for induction and maintenance of remission. An open-label study showed significantly decreased disease activity and increased mucosal alkaline sphingomyelinase activity, which, through hydrolysis of sphingomyelin and resultant ceramide production, induces epithelial differentiation and apoptosis and can inhibit proliferation (24). Another study found that VSL#3 treatment reduced levels of platelet-activating factor, a pro-inflammatory compound implicated in the development and activity of IBD (25). A randomized controlled trial in the pediatric population observed an effect on both induction and maintenance of remission over a 1-year period, with significantly lower endoscopic and histologic measures of disease activity in the treatment group even at time of relapse (26). When administered in combination with VSL#3, the 5-ASA prodrug balsalazide, formulated for targeted colonic release of 5-ASA, was superior to either balsalazide monotherapy or traditional 5-ASA in inducing remission (27). Administration of VSL#3 alone resulted in significant improvement in both disease parameters and remission rate compared to placebo (28). A second VSL#3 study also observed a significant decrease in disease activity, although the remission rate was not significantly improved compared to placebo (29). Finally, a study explored the immunomodulatory effects of the mixture by comparing the probiotic to corticosteroid treatment, placebo-treated patients, and healthy controls. The authors observed that both corticosteroids and VSL#3 enhanced IL-10 expression in dendritic cells (DCs) and reduced expression of TLR-2 and IL-12, although VSL#3 patients showed a non-significant improvement in clinical responses (30).

Other probiotic mixtures have been evaluated in UC with varying success. A randomized placebo-controlled trial studying the effects of bifidobacteria-fermented milk on UC observed improvement in disease activity, which was associated with significant increases in fecal butyrate, propionate, and short-chain fatty acid concentrations in the treatment group compared to the placebo group (31). A mix of *Bifidobacterium* species was found to positively influence relapse rate and demonstrated a variety of anti-inflammatory effects (32). Treatment with a mixture of *Lactobacillus delbrueckii* and *Lactobacillus fermentum* significantly reduced mucosal leukocyte infiltration. Expression of IL-6, TNF-α, and NF-κB and fecal calprotectin levels were shown to be associated with neutrophil infiltration of intestinal tissues when compared to the placebo treatment (33). In contrast, a trial of a mixture of *Lactobacillus acidophilus* and *Bifidobacterium animalis* subsp. *lactis*, in which no other concomitant UC treatments were permitted, found no significant clinical benefit (34).

### Probiotics and Pouchitis

Colectomy is a common surgical treatment modality for UC, after which pouchitis, or inflammation of the surgically created ileal pouch, is a common complication. Pouchitis is clinically associated with frequent relapses and appears to be linked to intestinal bacterial composition given the results of several probiotic studies (35).

Mixed probiotic agents, including VSL#3, have been studied for efficacy in secondary prevention of pouchitis (36). Among patients who underwent probiotic treatment to maintain antibiotic-induced remission, only 15% relapsed compared to all patients in the control group (*p* < 0.001). Microbial components of the supplement could be detected in fecal samples during the trial period, but returned to baseline levels within a month of discontinuation, and all remissions relapsed within 4 months after completing treatment. These findings were supported by a similar study of patients with chronic or relapsing pouchitis (37). VSL#3 was also found to prolong time before onset of primary pouchitis, which was again correlated with an increase in fecal colonization of the probiotic species (38). Treatment has been associated with an increase in regulatory T-cells and a decrease in expression of pro-inflammatory cytokines in the intestinal mucosa of treated subjects (39). However, a study of long-term remission maintenance, modeling more realistic use in actual clinical practice, found no benefit, and subclinical evidence of mild-to-moderate disease was found on endoscopy in the 19% who remained in the study for the full 8 months (40).

Other studies of mixed probiotics have been generally positive. One study evaluated a subset of VSL#3 components in treatment for pouchitis (41). After 9 months of probiotic treatment, the number of patients with pouchitis was reduced when compared to the control group, concluding that long-term probiotic use can serve as an effective method of pouchitis prevention. Another study found that a fermented milk product containing lactobacilli and bifidobacteria altered fecal microbiota composition and significantly improved superficial measures of pouchitis compared to the control (42).

Studies that focus on a single bacterial species have also been reported to have some efficacy for pouchitis. A small 6-month trial of *Bifidobacterium longum* found a significant difference in clinical, endoscopic, histological disease, and microbiota parameters (43). A small double-blind study of *Clostridium butyricum MIYAIRI* found a non-significant trend toward developing pouchitis in the placebo group, but endoscopic and microbial data were inconclusive (44). In contrast, administration of *Lactobacillus rhamnosus GG* to maintain pouchitis remission was ineffective despite shifts in microbial community profiles observed during treatment (45).

### Probiotics and CD

Compared to the large number of studies performed in patients with UC, less evidence exists to support the efficacy of either single-strain or multistrain probiotics in inducing or maintaining remission in patients with CD. Several small randomized controlled trials of *Saccharomyces boulardii* observed significant reduction in symptoms (46), significant effect on relapse rate (47), and decreased intestinal permeability (48); however, a 52-week trial of 165 patients found no effect of the yeast on either latency to relapse or relapse rate (49). VSL#3 was found in one study to display a trend toward benefit in maintenance of surgical remission, as well as significant reduction in IL-1β, TNF-α, and IFN-γ and increase in TGF-β, which correlated with decreased endoscopic disease activity (50). Several other studies of VSL#3, however, found no significant effect of the supplement on relapse rate or cytokine profiles (51–54) and one investigation found administration to be associated with an increase in symptom flares (55). Other trials of single-species probiotics were similarly unencouraging, with EcN administration over a 1-year maintenance period demonstrating a non-significant trend to benefit (56), and two large trials of *Lactobacillus johnsonii* demonstrating no effect on disease activity or recurrence (57, 58).

### Prebiotics and IBD

Dietary interventions designed to provide intestinal bacteria with metabolic substrates are termed prebiotics and can include fibers, resistant starches that are difficult for the small intestine to completely digest, and poorly absorbed monosaccharides, oligosaccharides, and polysaccharides. A study of CD patients asked subjects to quickly transition from a low-residue diet that is commonly recommended for IBD to a high-fiber diet rich in vegetables and found that all achieved disease remission within 2 months, which was sustained in 92% of patients at 2 years without scheduled maintenance pharmaceutical therapy (59). Another study found promising results with germinated barley foodstuff for maintenance of remission and potentially decreased steroid burden with reduced risk of relapse (60). In contrast, lactulose had no significant effect on clinical, endoscopic, or immunohistochemical parameters in either UC or CD (61). Fructooligosaccharide (FOS) supplementation, however, during active CD increased the abundance of fecal bifidobacteria and also led to increased secretion of IL-10 by intestinal DCs (62). A subsequent randomized, double-blind trial of FOS compared to a placebo for 4 weeks found a similar augmentation of IL-10 production by DCs, but unfortunately neither significant clinical benefit nor differences in fecal concentration of potentially beneficial commensals were found (63). Another study of FOS in combination with inulin found no change in inflammatory mediators IL-8 and PGE-2 or disease activity, but there was a significant reduction in calprotectin levels (64). Administration of single-agent inulin to patients with pouchitis reduced *Bacteroides fragilis* concentration in feces, increased fecal butyrate concentrations, and attenuated pouchitis disease activity index (65). Finally, prebiotic maintenance treatment of UC with *Plantago ovata*, also known as psyllium, was found to be more effective than placebo over 4 months (66), but was no better than traditional 5-ASA treatment in a long-term study of 1 year (67).

### Synbiotics and IBD

The results of trials with synbiotics, or matched combinations of probiotics and prebiotics, in the management of IBD have been inconsistent. In UC, a randomized controlled study of *B. longum* + FOS + inulin demonstrated no significant disease mitigation (68); however, bifidobacteria + galactooligosaccharide significantly reduced endoscopic disease parameters, as well as levels of fecal Bacteroidaceae (69). A 2-year study in CD patients on the efficacy of synbiotics for maintaining surgical remission found no effect on any disease parameters, although the trial ended prematurely due to poor enrollment (70).

### FMT and IBD

Fecal microbiota transplant has gained traction as a successful therapy for chronic, recurrent, or resistant *Clostridium difficile* infection. Although the pathophysiology of IBD is likely more complex than that of *C. difficile* infection, several case and cohort studies and two randomized controlled trials have begun the investigation of FMT as a treatment methodology. We have divided our summary of these studies into single-arm studies with either upper or lower introduction approaches, followed by controlled studies.

Single-arm studies of upper FMT introduction have been reported. Two studies in CD patients looked at the effects of nasogastric or gastroscopic FMT administration over a follow-up period of 1–3 months. FMT administration in a pediatric cohort induced remission within 2 weeks in 7 of 10 patients, all of whom demonstrated microbial engraftment, and remission was sustained by 5 patients at 6 weeks (71). However, a second pediatric study of an additional four patients by the same group found the same procedure to have no effect over the 3-month trial period (72). In a larger study of 30 patients receiving 1–3 treatments, clinical improvement and steroid-free maintenance were observed in 8 patients and a complete remission in another 4 patients (73). However, series of three nasojejunal transplants in four CD patients was associated with only transient changes in microbiota composition and no improvement in symptoms (74), while a second trial of nasojejunal in five UC patients reported only one instance of clinical improvement at the end of 12 weeks and the deterioration of two subjects within a month (75).

Single-arm studies of lower FMT introduction have also been reported for UC. Treatment with a series of five once-daily transplants induced remission that was sustained throughout the month-long trial period in three of nine subjects, and clinical response was observed in another five patients by the conclusion of the trial (76). A study of single transplants in 11 patients showed a benefit in both disease activity and quality of life measures at the end of 4 weeks (77). A case report of a patient with steroid-dependent UC who was treated shortly after primary onset of the disease reported that a series of 3 transplants 4–6 weeks apart led to complete mucosal healing (78), and a case series of 11 patients similarly observed reductions in disease activity in all 11 patients (79). In a trial of eight patients, two achieved remission after 2 weeks and another by the end of the 12-week period, while four others showed clinical improvement (80). Six of six patients demonstrated clinical improvement within 2 weeks of a single transplant, with sustained improvement at 3 months observed in two patients (81). The group also reported a shift in fecal microbial profile toward donor structure in three patients, although evidence of a shift did not correlate with symptomatic response. Another study of single transplants in UC patients surprisingly noted no correlation between microbial engraftment and clinical response to therapy, but nevertheless observed response in three of five subjects (82). A subsequent 3-month study by the same group noted remission in only one of six patients at the 1-month time point, and all patients displayed worsening symptoms by the end of the trial (83). Again, achieving remission was not associated with success of donor flora engraftment; interestingly, however, loss of similarity achieved over time did correlate with the reemergence of disease activity. Finally, a single-institutional review of all FMTs administered to UC patients found that complete clinical remission was observed in 42 of 62 patients and partial response and failure in 15 and 5 patients, respectively (84). This group has also described a case of complete and unexpected reversal of concomitant immune-mediated thrombocytopenia, as well as gastrointestinal symptom attenuation, in a woman undergoing the procedure in an effort to control her chronic, relapsing disease (85).

Single-arm studies of lower FMT in CD patients have also been performed. Two single-transplant studies conducted showed preliminary promise for CD patients, with one showing remission in half of the 8 subjects after 2 months (86) and the other reporting response in 11 of 19 subjects (87), over half had maintained their stability at 12 months, although 7 experienced a worsening of symptoms. They also noted significant species-level similarity between donor and recipient in responders compared to non-responders, as well as increased microbial diversity. A small study of the long-term effects of colorectal FMT was carried out in 3 patients, who received a tapering course of 22–30 transplants over 6–12 weeks (88). All three achieved immunotherapy-free remission for over 2 months until symptom return motivated a return to conventional therapies.

Finally, two controlled studies of FMT for UC have been reported with opposing results. One trial randomized 75 UC patients to receive either 6 weekly FMTs or water enemas and found that those in the treatment group had a superior rate of achieving remission (89). Greater efficacy was seen in those patients who had been diagnosed within a year, perhaps indicating an association between outcome and degree of mucosal damage. Interestingly, the source of FMT may be an important factor, stool from one of six donors induced remission in nearly 40% of recipients, significantly higher than the 10% achieved by each of the other five donors. In contrast, a study of 48 patients randomized individuals to receive two nasoduodenal FMTs from either healthy donors or their own stool and found no significant differences between the treatment and placebo groups (90). The conflicting results of these two studies could be attributed to a number of differences in methodology, including site of administration (upper versus lower gastrointestinal tract introduction), type of control treatment, and number of treatments per patient, and so to date, the efficacy of FMT for UC and IBD in general remains to be fully elucidated.

Overall, clinical and microbial responses to FMT in patients with IBD appear to be mixed and when positive unfortunately are often transient. A handful of impressive clinical responses, however, have been reported. As the bulk of the studies thus far have been small, short, and uncontrolled, further research into optimal donor and patient characteristics, exploration of underlying immunomodulatory mechanisms, and substantiation of long-term efficacy are warranted.

## Circulatory

Many studies have investigated the role of microorganisms in the pathophysiology of vascular inflammatory conditions, including atherosclerosis (91, 92), aortic aneurysms (93), and systemic vasculitides such as Behçet’s syndrome (94) and polyarteritis nodosa (95). Several studies have identified bacteria within the diseased vasculature, primarily in the setting of atherosclerosis, while derangements in intestinal bacterial composition have been seen in patients with systemic vasculitis.

### Atherosclerosis

Inflammation is an important contributor to the pathogenesis of atherosclerosis. Thought to arise from endothelial dysfunction, the resulting inflammation in the form of an accumulation of macrophages leads to deposition of low-density lipoproteins and eventual formation of fatty streaks. A role for bacteria in the pathogenesis of atherosclerosis was identified when the respiratory tract pathogen *Chlamydia pneumoniae* was detected within atherosclerotic vessels (96). Interestingly, infection of macrophage precursors (monocytes) by *C. pneumoniae* increased their adherence to endothelium, and chlamydial lipopolysaccharide was considered a key player in this process (97). T cell responses against *C. pneumoniae* have also been demonstrated within atherosclerotic plaques (92), suggesting an additional potential contribution from the adaptive immune system. Besides *C. pneumoniae*, other bacteria such as *Porphyromonas gingivalis* have also been implicated in the inflammatory process that leads to atherosclerosis (91). More recently, the intestinal microbiota have been implicated in the pathogenesis of atherosclerosis by altering the metabolism of ingested dietary compounds. l-carnitine, a trimethylamine and a compound found in red meat, is metabolized by intestinal bacteria to trimethylamine *N*-oxide (TMAO), which was found in higher concentrations in non-vegan humans than in vegans (98). TMAO was found to enhance calcium release from platelets stores in response to various stimuli, thus increasing their thrombogenic activity and accelerating atherosclerosis in animal models (99).

### Vasculitis

Many systemic vascular diseases have been historically associated with a specific microorganism; an example is the link between polyarteritis nodosa and hepatitis C virus (95). Another systemic vasculitic disease, Behçet syndrome, may have an intestinal microbiota contribution to its pathogenesis. Patients with Behçet syndrome have a significant dysbiosis of their gut microbiota and also a decrease in the short-chain fatty acid bacterial metabolite butyrate compared to healthy controls (94). Giant cell arteritis has been found to be associated with several viruses and bacteria, but a recent unbiased DNA sequencing study of temporal artery biopsy samples was unable to confirm these associations (100).

## Integumentary

The skin is the largest organ in the body. In addition to providing barrier function, it also harbors a reservoir of numerous populations of microbiota. Recent advances in molecular approaches toward culturing microbiota on the skin have revealed a huge topographical variability of skin microbiota (101). Researchers have begun to examine this variability and determine whether it could contribute to a predilection for developing certain skin conditions at particular body sites, for example, psoriasis tends to affect hairy scalp and extensor surfaces, while eczema tends to affect flexor surfaces.

### Atopic Dermatitis (AD)

Atopic dermatitis, also known as eczema, is a common chronic inflammatory skin disease characterized by frequent flares of inflammation with subsequent pruritus, dryness, and scaling. Two mechanisms were historically hypothesized to explain eczema: one mechanism considers the trigger an epithelial barrier dysfunction secondary to an intrinsic defect in epithelial cells and the other mechanism suggests that IgE sensitization occurs secondary to an immunological disturbance and inflammation, which then leads to epithelial barrier dysfunction (102).

Recent studies have revealed a relationship between the severity of AD flares and the type of bacterial communities colonizing the skin and overall diversity (103). A longitudinal examination of antecubital and the popliteal regions of children with eczema found that diversity was significantly lower during the no-treatment flares, and *Staphylococcus aureus* was the predominant bacteria, while there was no significant difference in diversity during the baseline, intermittent treatment, postflare, and controls. These findings suggest that dysbiosis of skin microbiota occurs during AD, although whether this dysbiosis contributed to inflammation remains unclear. Interestingly, however, treatment of AD with topical steroids and antimicrobials resulted in restoration of bacterial diversity by decreasing *S. aureus* predominance in favor of *Streptococcus, Corynebacterium*, and *Propionibacterium* species.

The initial mechanisms or triggers that induce inflammation in AD are unknown. Some evidence suggests that AD may begin independently of IgE and that IgE-mediated sensitization occurs after the first lesion appears (102). Mast cell degranulation occurs after the development of antigen-specific IgE. *S. aureus* produces several toxins, including alpha toxin that targets cell membranes and induces mast cell degranulation, supporting a potential immunological link between *S. aureus* colonization and development of AD (104).

### Psoriasis

Psoriasis is an inflammatory skin disorder characterized by hyperproliferation of keratinocytes. This is thought to be a response to an unknown trigger with possible interactions between the host microbiota and the immune system (105). One study examined the difference in bacterial composition between psoriatic lesions, unaffected skin of psoriatic patients, and healthy participants using the 16S rDNA PCR on skin swabs (106). In contrast to normal participants and healthy skin samples of psoriatic patients where Actinobacteria (predominately *Propionibacterium acne*) were the most abundant and diverse, psoriatic lesions showed an abundance of Firmicutes such as *Streptococcus*. Similarly, Proteobacteria were detected less frequently in psoriatic lesions compared to healthy controls. A related study of skin biopsies found a similar loss of *Propionibacterium* and an increased abundance of *Streptococcus* and *Staphylococcus* in psoriatic lesions (107). However, unlike the swab study described above, Proteobacteria in biopsies were found at much higher levels in psoriatic lesions than in controls.

A weak association between development of psoriasis and preceding antibiotics has been reported, potentially indicating a link between microbiota perturbation and disease. However, in this study, a history of prior skin infections was also associated with having a diagnosis of disease regardless of whether patients received antibiotics, leading the authors to conclude that infections could be associated with the development of pediatric psoriasis but antibiotics do not appear to contribute substantially to that risk (108). An additional caveat includes the possibility that misdiagnoses of skin infections were occurring in children at times prior to the making of a clear diagnosis of psoriasis.

### Systemic Sclerosis (SSc) (Scleroderma)

The hallmark of SSc is pathological fibrosis of skin and internal organs, which is thought to arise from abnormalities in the vascular and immune system. An autoimmune disease that typically affects middle-aged women, SSc can also occur in men and children. Its pathogenesis is multifactorial and includes genetic, autoimmune, and environmental factors. The presence of autoantibodies further supports the autoimmunity aspect of this disease and are utilized in categorizing SSc into diffuse and limited forms. However, to date, there is no animal model of SSc that successfully induces the disease by immunization against a specific autoantigen or *via* transfer of immune cells. Thus, the role of the immune system in the pathogenesis of this disease remains unclear (109).

Infectious agents that have been previously shown to be associated with the pathogenesis of SSc include parvovirus B19, Epstein–Barr virus, endogenous retroviruses, *Helicobacter pylori*, and chlamydial species (110, 111). In addition, antibodies against cytomegalovirus, hepatitis B virus, and toxoplasmosis have been detected at higher frequencies in patients with SSc, suggesting a role for these infectious agents in the initiation or progression of disease (110). It remains unknown whether the association between SSc and infectious agents is causal or if, alternatively, the immune system in these patients is altered leading to increased exposure to microorganisms.

*Rhodotorula glutinis*, a member of the fungal microbiome, was found to be overrepresented in the skin of a subset of SSc patients with an early diffuse presentation subtype (112). An environmental yeast that is known to cause fibrosis in the setting of opportunistic infections such as dialysis-associated fungemia, *Rhodotorula* has been hypothesized to contribute to inflammation-mediated fibrosis in this subset of SSc patients.

Although neither the mechanisms nor the pathogens responsible for the development of SSc have been definitively proven (111), four potential mechanisms have been studied: (1) molecular mimicry that may induce antibody production against vascular antigens, (2) endothelial cell damage that could be mediated by the direct toxic effects of the microorganisms or as a reaction of immune response against them, (3) superantigens derived from bacteria, and (4) microchimerism, where the presence of cells or DNA can be found in an individual but originated from a different organism (most commonly a fetus), and patients with diffuse SSc have been found to harbor more CD4+ microchimeric T cells than controls (111).

## Musculoskeletal

### Rheumatoid Arthritis (RA)

Of the various arthritic diseases, a relationship with the microbiome has been best characterized in patients with RA. RA is a systemic disease that affects multiple organs at different stages. Thus, studies of dysbiosis of the microbiota in RA patients have focused on a variety of sites to better understand its multisystemic manifestations. One study examined the bacterial composition of oral (dental and salivary) and fecal samples in patients with RA in comparison to healthy controls (113). Notably, RA patients were noted to have both oral and fecal flora that were distinct from that of controls, characterized by reduced *Haemophilus* spp. and increased *Lactobacillus salivarius*. The increase in *L. salivarius* could be interpreted as consistent with a prior study that found increased and more diversity in bacteria from the *Lactobacillus* genus in RA patients, including *L. salivarius* (114). However, not all lactobacilli may be pro-inflammatory in the setting of RA, as an interventional study with *L. casei* capsules showed reduced inflammation and improved severity of disease in patients receiving the probiotic (115). RA patients also had generally an increase in Gram-positive bacteria in the intestinal tract compared to controls, as well as an increased in oral anaerobic bacteria. Interestingly, levels of anticitrullinated protein autoantibodies, which are used in the clinical diagnosis of RA, were positively correlated with oral *Actinomyces* spp. and negatively correlated with oral *Haemophilus* spp. (113).

*Porphyromonas gingivalis* has also been reported to be associated with RA severity, although results have been mixed. *P. gingivalis* DNA was significantly more likely to be found in the synovial fluid of patients with RA than in controls (116), and interestingly the severity of periodontal disease (quantified by the number of missing teeth) in patients with RA was positively correlated with the number of affected joints. However, another study found only an association between *P. gingivalis* and the likelihood of developing and severity of periodontitis but no association with RA (117), and a third study found that *P. gingivalis* was more abundant in the oral cavity of controls compared to cases (113).

*Prevotella copri* has been linked with disease activity in RA patients (118). It was found in increased abundance in fecal samples from untreated newly diagnosed RA patients, while both treated chronic RA patients and controls had reduced amounts. The study went on to identify *Prevotella*-derived genes that were also associated with disease activity and also demonstrated exacerbation of disease by *P. copri* in a murine model of colitis. Thus, intestinal *P. copri* appears to exacerbate inflammation and may be a viable target in patients with autoimmunity.

## Respiratory

### Asthma

Asthma has been described as a triad of airflow obstruction, bronchial hyperresponsiveness, and lower airway inflammation. The inflammatory trigger is thought to be multifactorial, although in genetically susceptible individuals, the immune system has been particularly implicated, specifically in the way it interacts with environment exposures (119). Both airway and intestinal sites of commensal bacteria have been associated with asthma outcomes. Respiratory airways are not sterile and in fact harbor significant populations of microbiota. Changes in the composition of airway bacteria have been found to be associated with asthma and emphysema, as well as with exacerbations of these diseases (120).

In a prospective cohort study, culture-based assays of the hypopharynx found that asymptomatic neonates at 1 month of age who were colonized with *Streptococcus pneumoniae, Hemophilus influenzae*, or *Moraxella catarrhalis*, or a combination of these organisms, were more likely to have manifestations of asthma at 5 years of age (121). *Hemophilus* and other Proteobacteria have also been implicated in studies comparing the airway flora of asthma patients with controls that utilized sequencing-based approaches (120, 122) and have been associated with more severe (corticosteroid-refractory) disease (123). Whether colonization with these organisms can help cause asthma, and potential mechanisms for how they may do this, remains to be fully explored.

The intestinal microbiome may also help contribute to asthma or other allergic diseases, particularly early life exposures to intestinal dysbiosis, although each study has had slight inconsistencies. In a large study of over 400 infants, intestinal flora diversity in the first year of life was associated with protection from development of allergic sensitization, allergic rhinitis, and peripheral blood eosinophilia, but not asthma or AD (124). In contrast, a smaller study of just fewer than 50 infants found that samples collected within 1 month of age showed that intestinal flora diversity was associated with protection from later development of asthma, but was not associated with protection from other allergic manifestations (125). A third recent study of over 300 children who were at a higher risk for developing asthma was able to identify 4 bacteria taxa that were associated with protection in the first 100 days of life: *Lachnospira, Veillonella, Faecalibacterium, and Rothia*. An examination of urinary and fecal metabolites found that reduced urinary urobilinogen and increased fecal acetate were also associated with protection, suggesting that bacterial involvement in the metabolism of bile and short-chain fatty acids could be contributing to modulation of asthmatic risk. Interestingly, administering a cocktail of bacteria from these taxa led to reduced disease severity in a gnotobiotic mouse model of asthma, providing additional evidence for potential causality (126).

## Neuromuscular

A potential interaction between the nervous system and the gut has only recently expanded to incorporate enteric flora (127). While the preponderance of current research is preclinical, knowledge of the gut–brain axis and the underlying neurologic, immunologic, and endocrine processes that facilitate communication between the two distal sites, as well an increasing understanding of the central role of the microbiome in intraintestinal and extraintestinal immunity, have indicated probable microbial involvement in human neuromuscular disorders such as multiple sclerosis (MS), Guillain–Barré syndrome (GBS), and fibromyalgia (FM).

Both MS and GBS are demyelinating diseases; MS is characterized by a vast range of neurological symptoms stemming from damage to the central nervous system (CNS) (128) and GBS presenting as rapid-onset sensory changes and ascending muscle weakness as a result of damage to peripheral nerve cells (129). Although MS has also been linked to genetic variation (130), both MS and GBS are thought to occur in large part due to environmentally acquired triggers that catalyze autoimmunity (128, 129). However, a murine model of MS has demonstrated a role for commensal enteric flora to drive T cell responses against a myelin autoantigen in gut-associated lymphoid tissue, which then condition and drive proliferation of antigen-specific B cells, resulting in spontaneous autoimmune demyelination (131). This demonstration of the immunomodulatory contribution of the gut flora in the pathogenesis of CNS disorders, as well as clinical evidence of dysbiosis or infection associated with MS and GBS, supports exploration of potential prognostic biomarkers in the microbiota as well as the effects of therapeutic microbial intervention.

### Multiple Sclerosis

Compared to healthy controls, the flora of patients with MS is notable for a relative decrease in bacteria belonging to Clostridia and Bacteroidetes (132, 133), as well as an overall decrease in species richness in relapse-remitting (RRMS) patients. RRMS was further associated with significant intestinal permeability as measured by lactulose/mannitol urinary ratio (134). A different study of vitamin D therapy for MS found an increase in abundance of *Akkermansia, Faecalibacterium*, and *Coprococcus* genera in patients with previously untreated MS (135). Because strains of *Faecalibacterium* and *Coprococcus* are producers of the anti-inflammatory short-chain fatty acid butyrate, and experimental colonization with *Akkermansia* implicated members of the genera in immune tolerance of commensal gut microbes, these results suggested that intestinal bacterial shifts could help mediate beneficial effects of vitamin D therapy in MS patients. Intestinal dysbiosis has also been associated with modulation of gene expression in several immune cell populations, including maturation of DCs and activation of T cell and monocytes (136). A pediatric study of patients within 2 years of primary disease onset observed two associations between abundance of a bacterial subset and an immune parameter that were seen in the control cohort but not the case population (137). *Fusobacteria* was strongly positively associated with Tregs, and Firmicutes abundance was inversely associated with pro-inflammatory Th1. A prior study by the same group observed a significant inverse relationship between *Fusobacteria* abundance and MS relapse risk (138).

Pathogenic bacteria have also been reported to be associated with development or maintenance of MS. In one patient, *Clostridium perfringens* type B colonization could be detected shortly after primary disease onset and was associated with actively enhancing lesions on brain MRI (139). The group found evidence that epsilon toxin (ETX), secreted by *C. perfringens*, disrupts the blood–brain barrier and selectively binds to CNS myelin and white matter, which supports its involvement in lesion formation. Although identified only in a small minority of samples, possibly due to known difficulties in maintaining humoral ETX immunity, reactivity to ETX was found to be 10 times more prevalent in patients with MS versus healthy controls. While this is the only known report of *C. perfringens* type B in a human, the human commensal type A was found in significantly more healthy controls than in MS patients, suggesting that the absence of this commensal could lead to an open ecological niche for non-commensal *C. perfringens* toxinotypes.

A few studies have reported efforts at microbial modulation in the treatment of MS. One three-patient series found that FMT performed to address MS-associated constipation resulted in progressive improvement of neurological symptoms and eventual sustained remission (140). Two small studies treated RRMS orally with ova from the non-pathogenic helminth *Trichuris suis* (141, 142), which has been successfully applied in cases of IBD. Both studies observed that *T. suis* was associated with an increase in anti-inflammatory and immunoregulatory serum cytokines and a decrease in MRI-detectable brain lesions. In addition to its documented immunomodulatory effects, helminth colonization was also associated with greater fecal species richness in an otherwise healthy population (143), although the microbiome was not examined in these trials. This is evidence that the correlated clinical improvement could be effected directly by the organism or indirectly by alterations in community structure and humoral immunity.

### Guillain–Barré Syndrome

The presence of pathogenic bacteria at disease onset is particularly associated with GBS, in which approximately 30–40% of patients show serological evidence of recent infection with *Campylobacter jejuni* (144–148). Many *C. jejuni*-positive patients demonstrate signs of enteric infection just prior to onset of neuropathy with the median interval of 9 days to onset of neurological symptoms (147). The latency period suggests that GBS may not be the direct effect of the pathogen or its toxins but rather could result from a mounted immune response against its presence. Current understanding of infection-associated dysbiosis (149) thus lends additional support to the role of the microbiota in demyelinating diseases.

### Fibromyalgia

The etiology of FM is less understood, but hypothesized to involve aberrations in the processing of pain signals, resulting in chronic diffuse pain and stiffness (150). An increase in serum IL-6 and IL-8 has been reported in FM patients, the concentrations of which were positively correlated to duration of disease (151). Although no comparisons of healthy and FM microbial community structure have been reported, intestinal permeability was significantly increased compared to controls in a cohort of 40 FM patients (*p* < 0.0003), suggesting that luminal antigens may be able to access and modulate immunocompetent cells as seen in the hepatic disorders detailed above (152). Furthermore, FM is often comorbid with IBD (153), IBS (154–158), and SIBO (158). Lactulose breath test administration to diagnose SIBO demonstrated abnormal results more commonly in FM patients than in controls, and the degree of somatic pain was significantly correlated with breath test hydrogen level, supporting further exploration of the dynamics of the enteric flora and FM.

## Systemic

### Systemic Lupus Erythematosus (SLE)

The hypothesized pathogenesis of SLE is similar to that of the other autoimmune diseases discussed herein, with genetic, hormonal, endocrine, and environmental factors all thought to play a role (159). SLE is associated with the largest number of detectable antibodies, resulting in the involvement of nearly every organ and a diversity of clinical manifestations (160). SLE-associated dysbiosis has been characterized in a few studies, highlighting changes in community structure, metabolics, and gene expression. SLE patients have been noted to have a relative increase in intestinal Bacteroidetes, resulting in a significant decrease in the Firmicutes/Bacteroidetes ratio (161). This was associated with a functional increase along oxidative phosphorylation pathways, suggesting possible compensatory adaptations to intestinal oxidative stress underlying the observed changes in community structure. Intestinal metabolomic analysis of SLE patients compared to controls found alterations in mediators of cell signaling, quorum sensing, and cell wall synthesis (162), and it was recently reported that dysbiotic microbiota from SLE patients elicited different *in vitro* immune responses from naive CD4+ lymphocytes than exposure to microbiota from healthy controls, promoting greater lymphocyte activation and Th17 differentiation (163). This response has the potential to exacerbate and sustain preexisting inflammation linked to the disease, which could therefore be mitigated by restoration of a balanced flora.

### Graft-versus-Host Disease (GVHD)

Graft-versus-host disease is an immune-mediated condition that commonly occurs in patients who have undergone allogeneic hematopoietic cell transplantation (HSCT), a procedure that can mediate curative long-term remissions for many hematological malignancies as well as benign hematological conditions. The presentation of GVHD can be largely categorized into two forms, acute and chronic, with acute GVHD often occurring within the first 3 months and typically affecting the skin, liver, gastrointestinal, and hematopoietic systems, while chronic GVHD typically occurs beyond the first 3 months and can present with a variety of manifestations including exocrine dysfunction and systemic fibrosis. Whether chronic GVHD may be associated with changes in the microbiome has not been well investigated. Acute GVHD, however, has long been thought to be strongly modulated by the microbiome, particularly intestinal bacteria. Based on animal studies that indicated intestinal bacteria contributed to acute GVHD, a randomized study in the early 1980s of protective isolation including gut-decontaminating antibiotics and skin disinfection showed a benefit with reduced acute GVHD (164). However, later studies did not reproduce this finding, possibly due to inconsistent success of gut decontamination (165). As a result, improved pharmacologic prevention of GVHD, particularly with calcinuerin inhibitors, has largely replaced gut decontamination for GVHD prophylaxis, though one clinical trial did demonstrate a benefit for suppressing intestinal obligate anaerobes with metronidazole (166). More recent efforts have re-examined the relationship between intestinal bacteria and acute GVHD using culture-free methods such as 16S deep sequencing. Development of acute GVHD produces striking changes in patient intestinal bacterial composition (167–170). Furthermore, microbiota damage either prior to HSCT (169) or early post-HSCT (171, 172) may increase the subsequent risk of developing GVHD. Much of this microbiota damage is manifested as loss of *Blautia* and other Clostridia (171, 172) or loss of Bacteroidetes (169). Perhaps not surprisingly, antibiotics are a common cause of microbiota damage in this patient population, and treatment with piperacillin-tazobactam (173), carbapenems (173), metronidazole (174), and clindamycin (170) have all been identified as associated with increased acute GVHD. Given these results, interest is building in targeting the microbiota for either the prophylaxis or treatment GVHD. A study of daily *Lactobacillus plantarum* administration in pediatric HSCT patients was found to be safe and well tolerated, even in the setting of neutropenia. In addition, a study of fecal transplantation as therapy for acute GVHD was recently reported, with three of four patients have a complete response and the last patient having a partial response, an overall promising result in the steroid-refractory setting, which typically has a poor prognosis (175).

## Conclusion

The causes of inflammatory diseases are multifactorial and include age, genetics, and environment. Microorganisms are crucial in maintaining gastrointestinal homeostasis and can potently modulate systemic immunity, and differences in the microbiota have been observed in patients with inflammatory diseases compared to healthy controls. There is a growing amount of clinical research being done to better understand the role that the microbial community can play in inflammatory diseases, and some progress has been made evaluating the effects of targeting the microbiome, particularly in the setting of IBD. Some intriguing responses suggest that this may be a viable strategy, but conflicting and inconsistent results leave open questions. One possibility may be that patient disease heterogeneity is a source of statistical noise, and it is likely that in a subset of patients with inflammatory conditions, alterations in the microbiome contribute significantly to drive disease pathogenesis and maintenance, while for other patients, the microbiome may be only a minor contributing factor. Developing strategies to distinguish these patient subsets will likely be critical to advancing the growing field of clinically targeting the microbiome for patients with inflammatory diseases.

## Author Contributions

AES, ZS, and RRJ wrote the manuscript. DHW prepared the table. All authors revised the manuscript and approved it for publication. AES and ZS have contributed equally to this work.

## Conflict of Interest Statement

RRJ is on the board of directors or an advisory committee for Seres Therapeutics, Inc.; has consulted for Ziopharm Oncology; and holds patents with or receives royalties from Seres Therapeutics, Inc. The other authors declare no conflict of interest.
